# Impact of Single Hemodialysis Treatment on immune Cell Subpopulations

**DOI:** 10.3390/jcm12093107

**Published:** 2023-04-25

**Authors:** Chiara Donadei, Andrea Angeletti, Valeria Pizzuti, Fulvia Zappulo, Diletta Conte, Maria Cappuccilli, Anna Laura Chiocchini, Anna Scrivo, Delia Apuzzo, Maria Addolorata Mariggiò, Lorenzo Gasperoni, Gabriele Donati, Gaetano La Manna

**Affiliations:** 1 Nephrology, Dialysis and Renal Transplant Unit, IRCCS-Azienda Ospedaliero-Universitaria di Bologna, 40139 Bologna, Italy; chiara.donadei@studio.unibo.it (C.D.); fulvia.zappulo2@unibo.it (F.Z.); annalaura.chiocchini@aosp.bo.it (A.L.C.); delia.apuzzo@studio.unibo.it (D.A.); lorenzo.gasperoni@gmail.com (L.G.); 2 Division of Nephrology, Dialysis and Transplantation, IRCCS Istituto Giannina Gaslini, Genoa Largo Gaslini, 16148 Genoa, Italy; andreaangeletti@gaslini.org; 3 Department of Medical and Surgical Sciences (DIMEC), University of Bologna, 40126 Bologna, Italy; valeria.pizzuti3@unibo.it (V.P.); diletta.conte2@unibo.it (D.C.); maria.cappuccilli@unibo.it (M.C.); 4 Department of Precision and Regenerative Medicine and Area Jonica, School of Medicine, University of Bari, 70121 Bari, Italy; mariaaddolorata.mariggio@uniba.it; 5 Nephrology and Dialysis Unit, Infermi Hospital, AUSL Romagna, 47923 Rimini, Italy; 6 Nephrology, Dialysis and Renal Transplant Unit, Azienda Ospedaliero Universitaria di Modena, 41124 Modena, Italy; 7 Surgical, Medical, Dental and Morphological Sciences Department (CHIMOMO), University of Modena and Reggio Emilia, 41124 Modena, Italy

**Keywords:** hemodialysis, immune response, inflammation, lymphocytes, Th1, Th2, IFN-gamma, Th17, B cells, natural killer cells, Annexin V, proliferation

## Abstract

Hemodialysis (HD) is known to trigger a chronic inflammatory status, affecting the innate and acquired immune response. This study was aimed at a comparative analysis of immune cell subsets, proliferation, and apoptosis in subjects receiving chronic HD treatment with respect to a healthy control. Regardless of the dialysis filter used, we observed a reshaping of the acquired immune component both with respect to healthy patients and between the various sessions of dialysis treatment, with an impairment of CD3 cells, along with an increase in CD4 and CD8 cell populations producing pro-inflammatory factors such as IL-17 and IFN-gamma. The population of B cells, monocytes and NK cells were not impaired by the dialysis procedure. These results confirmed the high impact of the HD treatment on the patient’s immune system, underlying the imbalance of T cell counterparts.

## 1. Introduction

Subjects affected by end stage kidney disease (ESKD) are characterized by worse comorbidity profile and higher mortality rates compared to the general population [1], mainly due to the increased risk of cardiovascular disease [2] (pp. 51–66), and of other complications related to uremia-induced immune dysfunction [3] (p. 742419), such as infections and neoplasms [4,5] (pp. S183–S187), (pp. 291–299).

Dialysis or kidney transplant represent the treatment of choice for subjects affected by ESKD. In particular, hemodialysis (HD) is an artificial procedure consisting of the partial replacement of kidney functions by an extracorporeal circulation of blood through an artificial kidney, which allows the removal of metabolic waste molecules and fluids. Such treatment is usually administered three times a week for several hours per session. Therefore, HD implies a continuous interaction between blood and artificial materials [6] (pp. 484–494).

Patients affected by ESKD receiving chronic HD usually present immune disorders that involve both innate and adaptive immune systems. Such comorbidities may be in part explained by the increased oxidative stress, endothelial dysfunction and chronic inflammation [7] (pp. 3963–3967), due to, among other things, the prolonged exposure of blood to the artificial HD components [8] (pp. S274–S280). In last two decades, the introduction of more biocompatible synthetic materials, such as polyacrylonitrile, an acrylonitrile-sodium methyl sulfonate copolymer, has significantly improved the efficacy of treatment and reduced the immunoreactivity against the HD filters compared to the previous membranes of cuprophane, a copper-substituted cellulose [9] (p. 71).

Despite the increasing availability of biocompatible materials, HD patients are still characterized by a chronic inflammatory status that promotes the early occurrence of immune dysregulation [8] (pp. S274–S280). However, the exact interactions between the immune system and HD membranes are still far from completely elucidated. Previous studies have reported the presence of several immune dysregulations, including strong activation of the complement pathways and an increased production of effector molecules, such as cytokines, interleukins and chemokines. These alterations elicit the recruitment and activation of immune cells, especially neutrophils and monocytes in subjects receiving chronic HD compared to the general population [10,11] (pp. 770–778) (pp. 82–94). Overall, the alteration of T-cells in HD negatively affects B cell response [12] (pp. 579–588), enhancing the risk of infections [13] (pp. 151–154). In view of this, a better awareness of the immunomodulatory effect of even a single HD session is essential to achieve successful dialysis treatment. The present study was undertaken to investigate the effect of a single hemodialysis treatment on the innate and adaptive immune system of ESKD patients.

## 2. Materials and Methods

### 2.1. Patients Characteristics and Study Design

This is an observational single-center analysis on patients receiving chronic hemodialytic treatment at the Unit of Nephrology, Dialysis and Transplantation of IRCCS University Hospital of Bologna, Italy. Inclusion criteria were age > 18 years, absence of residual diuresis, dialysis age at least of 1 year and arteriovenous fistula with blood flow > 250 mL/min. All patients received alpha erythropoietin with the same protocol. EPO was administrated intravenously at the end of each dialysis session with the reinfusion solution. Patients receiving blood transfusion in the previous month have been excluded from the study. For comparative analysis, serum samples of healthy subjects stored at the biobank of our Institute were used. In the HD population, blood samples were collected at 3 different time points: T0 and T1 corresponding to the beginning and the end of HD treatment, respectively, and T2 corresponding to the beginning of the next dialytic session, after 48 h. The study was conducted according to the guidelines of the Declaration of Helsinki and approved by the Local Ethics Committee (CE-AVEC number: 152/2019/Oss/AOUBo). Patients’ personal data were fully anonymized, and each subject patient was identified through a univocal code.

### 2.2. Hemodialysis Technique

Patients enrolled received three different HD treatments: HFR technique with HFR17 filter (Bellco, Mirandola, Italy), high-flow bicarbonate HD with BGU 1.6 Filtryzer filter (Toray, Tokyo, Japan) and bicarbonate HD with THERANOVA medium cut-off filter (Baxter, Heichingen, Germany). In detail, these comprised (i) HFR17 (Bellco, Mirandola, Italy), a double-chamber filter used for online HFR. The first part of the filter consists of a high-flux polyphenylene hemofilter with a Kuf of 28 mL/h/mmHg, a surface area of 0.7 m^2^ and a membrane cut-off value of 35,000 daltons. The ultrafiltrate then passes to a neutral styrene resin of 40 g which allows for an adsorbent area of 28,000 m^2^. After adsorption, the ultrafiltrate is added to the whole blood which, in turn, passes into the second HFR17 low-flow polyphenylene filter (Kuf 13 mL/h/mmHg, surface area 1.7 m^2^). (ii) Filtryzer BGU 1.6 (Toray, Tokyo, Japan) has a membrane of polymethyl methacrylate or PMMA, high flux (Kuf of 26 mL/h/mmHg), a surface area of 1.6 m^2^ and cut-off of 20,000 daltons. (iii) THERANOVA (Baxter, Heichingen, Germany) has a membrane of polyarylethersulfone and polyvinylpyrrolidone, a surface area of 1.7 m^2^, steam sterilization, a cut-off value of 25,000 daltons and a Kuf of 48 mL/h/mmHg.

### 2.3. Isolation of Peripheral Blood Mononucleate Cells

Peripheral blood mononucleate cells (PBMCs) were obtained from blood samples of healthy volunteers and HD patients according to the protocol approved by the Ethics Committee. Blood samples consisted of about 20 mL of peripheral blood collected in Vacutainer tubes containing Ethane-1,2-diyldinitrilo tetra-acetic acid (EDTA). After collection, peripheral blood was diluted in a 1:1 ratio with Phosphate Buffer Saline (PBS, Corning, NY, USA) containing 1% penicillin–streptomycin (P/S) solution (10,000 U/mL penicillin, 10,000 U/mL Streptomycin, Corning, NY, USA). PBMCs were isolated from peripheral blood via the density gradient centrifugation in Ficoll Paque (GE Healthcare Bio-Sciences AB, Uppsala, Sweden). Then, the stratified blood was centrifuged at 1500 rpm for 30 min without a break, and the ring containing the PBMC population was collected and resuspended in PBS. The cell suspension was again centrifuged at 1500 rpm for 10 min and the washing step was repeated twice. Isolated cells were frozen at −80 °C in a frozen medium containing 10% dimethyl sulfoxide (DMSO) until analysis.

### 2.4. Flow Cytometry Analysis

PBMCs from control and HD groups were characterized by flow cytometry. For the analysis of surface markers, cells were washed with PBS containing 0.1% Bovine Serum Albumin (BSA) and then incubated for 30 min at 4 °C with the following antibodies: CD4-APC (Biolegend, Cat. No. 300514), CD8-PerCP (BD, Cat. No. 345774), CD25-FITC (Biolegend, Cat. No. 302604), multitest CD3-FITC/CD16-PE/CD56-PE/CD45-PerCP/CD19-APC (BD, Cat. No. 340500) and simultest CD57-FITC/CD8-PE (BD, Cat. No. 333191). For the quantification of intracellular cytokines in lymphocyte subsets, a fraction of PBMCs was treated with a mixture of 1 nM Phorbol 12-myristate 13-acetate (PMA, Sigma Aldrich, St. Louis, MO, USA), Ionomycin 3 mg/mL (Sigma Aldrich, St. Louis, MO, USA) and 1 μL/mL Golgi PlugTM (BD, Becton, Dickinson, NJ, USA) and incubated at 37 ºC to 5% CO_2_ for four hours before staining. The intracellular staining was performed, after fixation and permeabilization steps, for 30 min in ice with the following antibodies: IFN-ꙋ-PECy7 (Biolegend, Cat. No. 502528) for the identification of CD4^+^IFN-ꙋ^+^ cells (Th1), IL17A-PE (Biolegend, Cat. No. 512306) for the identification of CD4^+^IL-17^+^ (Th17) cells and Foxp3-PE (Biolegend, Cat. No. 320108) for the identification of CD4^+^CD25^+^Foxp3^+^ (Treg) cells. For the proliferation analysis, the Ki67 FITC (BD, Cat. No. 556026) antibody was used. For all the experiments, unstained cells were used as negative controls. To reduce the impact of hemoconcentration and hemodilution on flow cytometry analysis, an amount of 100,000 events in the FSC-SSC gate were acquired for all time points, using a Cytoflex S (Beckman Coulter) flow cytometer equipped with 3 active lasers (405 nm, 488 nm, 561 nm) and 6 channels for fluorescence detection. Data analysis was performed using a dedicated software, Cytobank, Inc. (Beckman Coulter, Brea, CA, USA).

### 2.5. Cell Proliferation Analysis

PBMC proliferation at each time point was analyzed by evaluating the decrease in carboxyfluorescein succinimidyl ester (CFSE) fluorescence intensity (BD, Cat No. 51-9010817). Cells were thawed and resuspended in 5 mL of PBS with CFSE (2 µM) for 5 min at 37 °C. Reaction was blocked using a medium with 50% Fetal Bovine Serum (FBS, Gibco, Life Technologies, (Carlsbad, CA, USA). Cells were resuspended in culture medium RPMI 1640 (Corning) supplemented with 2 mm L-glutamine (EuroClone, Cat ECB3000D), 1% human serum AB (EuroClone, Cat ECS0219D), 100 U/mL penicillin–streptomycin (Aurogene, Cat AU-L0022-100), 0.1% β-Mercaptoethanol (Gibco, Cat 31350-010), 1% sodium pyruvate (Aurogene, Cat AU-L0642-500), 1% non-essential amino acids (Aurogene, AU-X0557-100). Cells were activated by anti-CD3 (Invitrogen, Cat 16-0039-85) and anti-CD28 (Invitrogen, Cat 16-0289-85) antibodies, and incubated for three days in 96-well plates at 37 °C and 5% CO_2_. At the end of the incubation period, PBMCs were collected and stained with anti-CD4 and anti-CD8 antibodies as described in the previous section. CFSE dilution in CD4 and CD8 gates was measured through the Cytoflex S flow cytometer and data were analyzed with the Cytobank software version 2.4.

### 2.6. Annexin V with 7-Aminoactinomycin D (7-AAD) Assay

Annexin V/7-AAD assay allows the detection of the early and late stage of apoptosis and the distinction between apoptosis and necrosis by flow cytometry. Thawed PBMCs were labelled with anti-CD3 antibody, as previously described, then washed twice and resuspended in 100 μL of binding buffer containing 2 μL of Annexin V conjugate PE (Biolegend, Cat 640908) and 7-AAD+ (Biolegend, Cat 79993). According to manufacturer instructions, cells were incubated for 15 min at room temperature and then diluted with 400 μL of binding buffer. Stained cells were analyzed for Annexin/7-AAD among the CD3 gate.

### 2.7. Statistical Analysis

GraphPad Prism (version 5 for Windows; GraphPad Software, Inc., Boston, MA, USA) was used. Based on the Shapiro–Wilk and/or Kolmogorov–Smirnov test, continuous variables were checked for normality of value distribution. Variables with normally distributed values were expressed as mean ± SEM, while variables with non-normal distribution of values were expressed as median with interquartile ranges. Student’s *t* test for independent samples, one-way ANOVA, and Kruskal–Wallis test were performed to estimate differences between groups, according to variable distribution. Statistical significance was set at *p* < 0.05.

## 3. Results

### 3.1. Demographic and Dialysis-Related Characteristics

Overall, 35 subjects receiving chronic hemodialysis (HD) treatment were investigated: of them, 12 were treated with Filtryzer BGU1.6, 12 with Theranova and 11 with HFR 17. The comparisons of patients treated with the three different dialyzers revealed similar demographics (age at inclusion and gender distribution) and inflammatory parameters (Table 1). Consistently, no significant differences in dialysis adequacy parameters were reported between the three groups (Table 2). In Table 3, dialysis efficiency parameters for the three dialyzers are reported. Serum samples of 10 healthy subjects were used as controls, matched for gender (women: *n* = 5, 50%) and age (72 years, 55–86 years).

The cell populations analyzed by flow cytometry did not show significant differences in the three dialysis groups. For this reason, the following results are presented as a single group.

### 3.2. Effects of HD Session on CD3^+^T-Cells and Apoptosis

**Figure 1:**
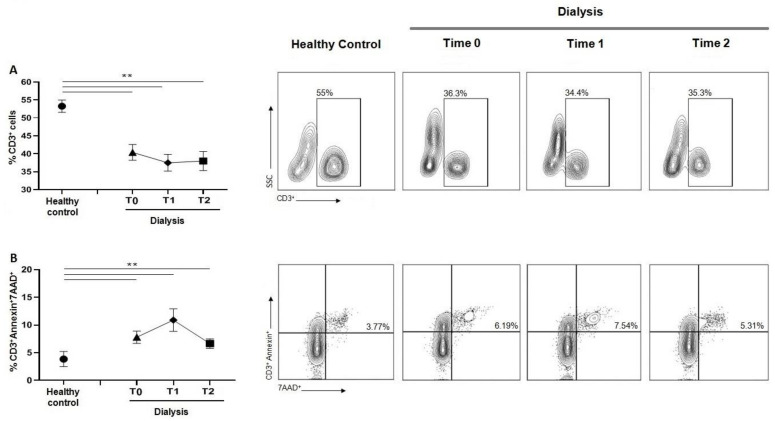
(**A**) Graph and related plots of CD3^+^ cells percentage among PBMCs of healthy controls (53.27 ± 5.41%) and during dialysis sessions: T0 (40.40 ± 2.27%), T1 (37.48 ± 2.35%), T2 (37.98 ± 2.66%). (**B**) Graph and related plots of apoptotic cells percentage among CD3^+^Annexin V^+^/7AAD^+^ in healthy controls (3.85 ± 1.37%) and during dialysis sessions: T0 (6.56 ± 1.15%), T1 (10.98 ± 2.02%), T2 (6.54 ± 0.86%). Statistical analysis was performed using *t* test, ** *p* < 0.01. Data points depict mean ± SEM.

The percentage of CD3^+^ T-lymphocytes in HD patients at T0 was significantly reduced in comparison to the healthy group. HD patients did not show significant changes in CD3^+^ cells over time, as the percentages were comparable at the three time points (Figure 1A). As shown in Figure 1B, a significant increase in apoptosis of CD3^+^ T lymphocytes was observed in HD patients at each time point when compared to healthy controls. The increase in Annexin V^+^/7AAD^+^ cells observed during HD session was not statistically significant.

### 3.3. Dialysis Session Increases the Proliferation Activity of CD4^+^ T and CD8^+^ Cells

**Figure 2:**
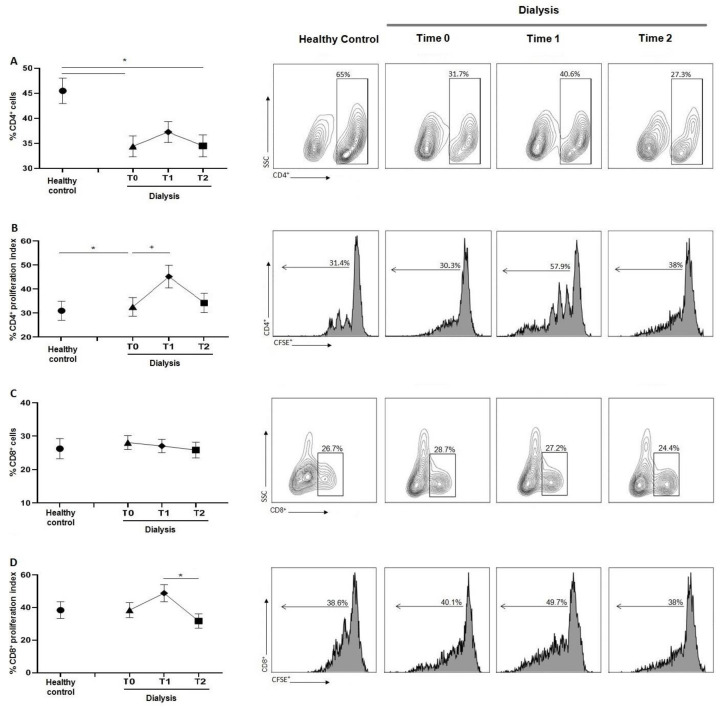
(**A**) Graph and related plots of CD4^+^ cells percentage among PBMCs of healthy controls (45.48 ± 2.55%) and during dialysis sessions: T0 (34.4 ± 2%), T1 (37.8 ± 2.08%), T2 (33.4 ± 2.66%). (**B**) Graph and related plots of CD4^+^ cells proliferation in healthy controls (31.01 ± 3.96%) and during dialysis sessions: T0 (32.63 ± 3.88%), T1 (45.26 ± 4.71%), T2 (34.27 ± 4.05%). (**C**) Graph and related plots of CD8^+^ cells percentage among PBMCs of healthy controls (28 ± 7.02%) and during dialysis sessions: T0 (28.07 ± 5.06%), T1 (27.2 ± 3.17%), T2 (24.4 ± 4.88%). (**D**) Graph and related plots of CD8 cell proliferation in healthy controls (38.9 ± 6.08%) and during dialysis sessions: T0 (40.1 ± 5.16%), T1 (48.8 ± 5.24%), T2 (31.75 ± 4.37%). Statistical analysis was performed using *t* test (* *p* < 0.05, Data points depict mean ± SEM.

As shown in Figure 2A, the percentage of CD4+ T cells was markedly reduced at T0 compared to healthy subjects and this depletion was maintained during dialysis sessions. However, despite the similar levels between healthy controls and HD patients, CD4 proliferation was significantly increased during dialysis sessions (Figure 2B). Concerning the proliferation rate analysis, there was a significant increase in CD4 proliferation at T1, compared with both control and T0, with a decrease at T2. Compared to controls, the dialytic treatment did not alter the percentage of CD8 T cells, as shown in Figure 2C. Regarding CD8 T cell proliferation, a reduction in proliferative cells was observed between T1 and T2, with no significative changes comparing to the controls (Figure 2D).

### 3.4. Chronic Dialysis Treatment Increases the Percentage of Th17 and Th1 Cells and Decreases the reg/Th17 Ratio

**Figure 3:**
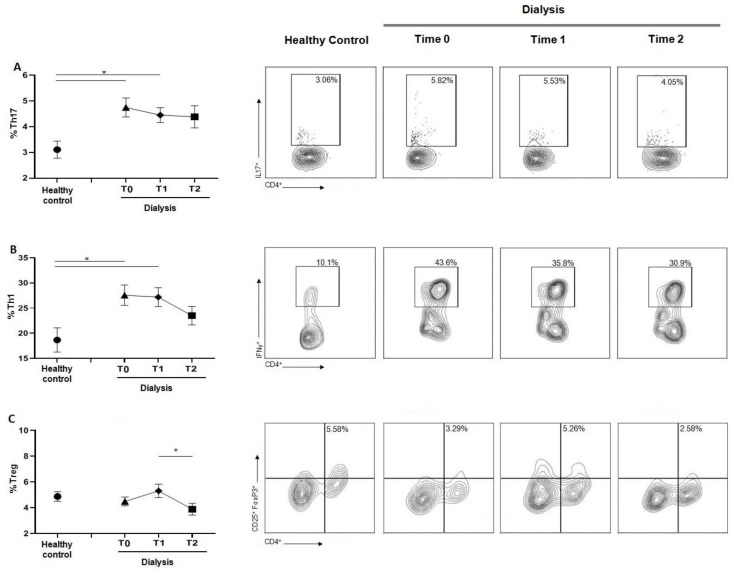
(**A**) Graph and related plots of Th17 (CD4+IL17+) percentage of healthy controls (3.11 ± 0.32%) and during dialysis sessions: T0 (4.75 ± 0.34%), T1 (4.45 ± 0.97%), T2 (4.39 ± 0.44%). (**B**) Graph and related plots of Th1 percentage (CD4+IFN-ꙋ+) of healthy controls (18.6 ± 2.46%) and during dialysis sessions: T0 (18.65 ± 2.49%), T1 (27.17 ± 1.86%), T2 (24.17 ± 3.63%). (**C**) Graph and related plots of Treg (CD4+CD25+FoxP3+) percentage of healthy controls (5.88 ± 3.52%) and during dialysis sessions: T0 (4.02 ± 1.67%), T1 (5.32 ± 0.55%), T2 (3.9 ± 0.47%). Statistical analysis was performed using *t* test, * *p* < 0.05. The + symbol indicates the statistical significance among T0, T1, T2 intra-dialysis time points, + *p* < 0.05.

As shown in Figure 3A, the Th17 subset significantly increased in dialyzed patients at T0 and T1 compared to controls. Despite a higher percentage of Th17 being reported at T2, statistical significance was not reached at this time point. Findings for Th1 (Figure 3B) cells were similar, with a significant increase at T0 and T1 compared to the healthy subjects. For both Th17 and Th1 populations, no significant changes during treatment were reported. The analysis of T regulatory (Treg) cells showed no significant difference in the percentage of Treg between patients on HD and healthy controls. Comparing the three time points, a significant difference in Treg value was observed between T1 and T2, with a marked decrease in their percentage at the last time point (Figure 3C).

**Figure 4:**
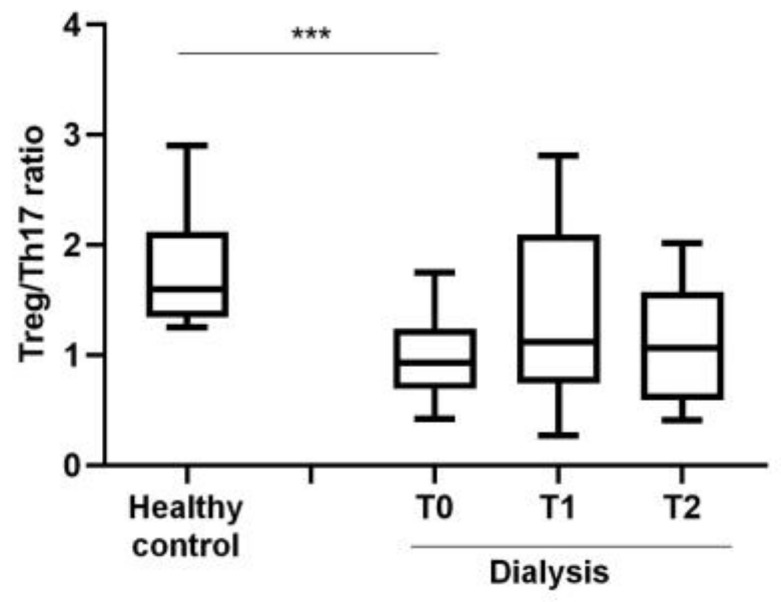
Evaluation of Treg/Th17 ratio in healthy controls and dialyzed patients. Statistical analysis was performed using *t* test, *** *p* < 0.001. Data points depict mean ± SEM.

The ratio Treg/Th17 was characterized by a significant decrease at every time point compared to healthy subjects (Figure 4).

### 3.5. Dialysis Session Reduces IFN-ꙋ Production by CD8 T Cells

**Figure 5:**
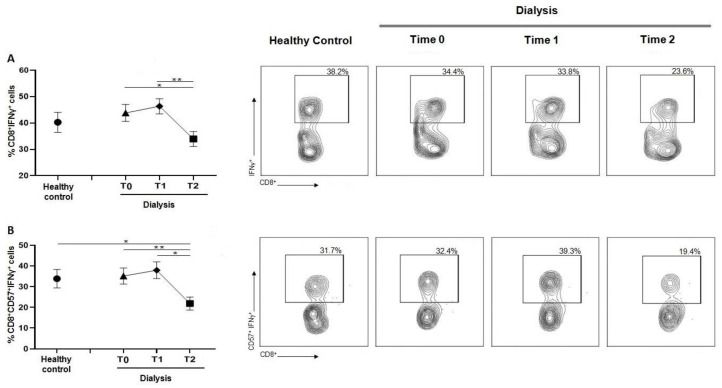
(**A**) Graph and related plots of CD8^+^IFN-ꙋ^+^ percentage of healthy controls (40.27 ± 3.78%) and during dialysis sessions: T0 (43.83 ± 3.22%), T1 (46.34 ± 2.88%), T2 (33.95 ± 2.82%). (**B**) Graph and related plots of CD8^+^CD57^+^IFN-ꙋ^+^ percentage of healthy controls (33.87 ± 4.46%) and during dialysis sessions: T0 (35.13 ± 3.85%), T1 (37.94 ± 4.01%), T2 (21.85 ± 3.14%). Statistical analysis was performed using *t* test, * *p* < 0.05, ** *p* < 0.01. Data points depict mean ± SEM.

As shown in Figure 5A, CD8^+^IFN-ꙋ^+^ was lower at T2 compared to the previous session (T1 and T2). Similar findings were reported for the CD8^+^CD57^+^IFN-ꙋ^+^ cells (Figure 5B), where a significant decrease was observed at T2, compared with both T0 and T1. This reduction in CD8^+^CD57^+^IFN-ꙋ^+^ was significant also if compared with healthy controls.

### 3.6. Dialysis Does Not Alter B Cells Content but Decreases the NK

**Figure 6:**
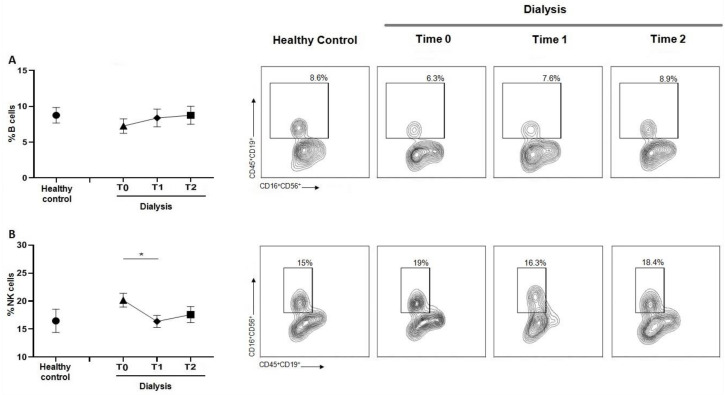
(**A**) Graph and related plots of B cells percentage of healthy controls (8.50 ± 3.66%) and during dialysis sessions: T0 (6.97 ± 2.96%), T1 (7.82 ± 4.51%), T2 (8.05 ± 2.90%). (**B**) Graph and related plots of NK percentage of healthy controls (16.68 ± 5.74%) and during dialysis sessions: T0 (20.7 ± 2.54%), T1 (17.05 ± 3.72%), T2 (18.72 ± 4.08%). Statistical analysis was performed using *t* test, * *p* < 0.05. Data points depict mean ± SEM.

The evaluation of the B cell population (Figure 6A) revealed that the percentage remained stable during the hemodialysis treatment, also compared with controls. Conversely, NK cells decreased during the session (Figure 6B).

## 4. Discussion

Subjects affected by ESKD are characterized by a chronic inflammatory status with an increased risk of mortality and morbidity [14] (p. 71). However, how hemodialysis (HD) may affect the immune system, favoring inflammation, is not completely known. In the present study, we investigated the immune response to a single session of HD, evaluating the alterations of the immune cells subset in subjects receiving chronic HD. We also compared the immunophenotype of such patients with heathy subjects.

The interaction between the blood and the extracorporeal components, such as membranes constituting the dialytic filters and lines, is one of the possible causes of systemic inflammatory status in HD patients, as demonstrated in several studies [5,8] (pp. S274–S280, pp. 291–299).

In this study, we observed a depletion of CD3 cells in patients receiving chronic HD compared to healthy subjects and the reduction of CD3 cells was associated with an increase in apoptosis. No significant changes were observed among the different HD sessions. Since CD3 represents a superficial T lymphocyte maturation marker, the decrease in CD3^+^ cells could reflect a reduced immune function [15] (pp. 581–585). Such a finding may justify the increased risk of infective disease that commonly characterizes HD subjects. The uremic and proinflammatory microenvironment in HD patients has been proposed as responsible for premature aging and apoptosis of immune cells [16] (pp. 208–217). In a previous report investigating the effect of the single HD session on T lymphocyte subsets in 14 subjects, Lisowska et al. [17] (pp. E77–E84) observed an increased CD4^+^/CD8^+^ ratio as a consequence of the reduction of CD8^+^ T cells and not of the increase in CD4^+^ T cells.

Consistently, we reported a reduction in CD4^+^ T cells and their proliferative activity at T0, compared to healthy volunteers, and confirmed the lymphopenia occurring during HD [18] (pp. 7–14). The increase in CD4^+^ T cells percentage and proliferation during the HD session may be explained as a temporary recovery of lymphocyte activity. Despite the decrease in CD4^+^ T cells percentage, we observed an increase in proinflammatory T cell subsets Th1 and Th17 compared to controls, without significant differences during HD session. The inflammatory microenvironment induced by the prolonged exposure to the HD components could promote the recruitment of proinflammatory cell populations that consequently enhance the inflammatory response compared to controls [19] (pp. 1070–1075). Another relevant sign of immune response alteration found in the HD group was the reduction of Treg/Th17 ratio, observed at T0. The differentiating shift in one subset to another strongly depends on the tissue cytokine environment, and the impairment in Treg could reduce tolerance, increasing the risk of self-response [20] (pp. 115–139). Prior data from our group did not highlight the immunological effect of erythropoietin (EPO) in counteracting T cell effects. EPO ligation of its receptor on CD4^+^ T cells prevented Th17 generation and induced trans-differentiation of Th17 into Foxp3^+^ CD4^+^ T cells, downregulating the pro-inflammatory T cell response [21] (pp. 2003–2015). Recombinant human EPO is usually administered at the end of HD treatment; therefore, it does not appear to represent a further variant in the modulation of the immune system in our cohort.

The evaluation of CD8 T cells and in particular their production of pro-inflammatory IFN-ꙋ is also an important hallmark of the immune system [22] (p. 310). While the percentage and proliferation of CD8 was not impaired by dialytic treatment, an increase in CD8 proliferation was observed at T1 compared with T0 and T2. The production of IFN-ꙋ by CD8 cells revealed that both CD8^+^IFN-ꙋ^+^ and CD8^+^CD57^+^IFN-ꙋ^+^ do not present significant differences between control and T0 or T1 dialysis time points. Interestingly, a significant reduction was observed at T2. These data suggest that the impairment of CD8^+^ cells, may result from the long-term effects of repeated hemodialysis [23] (pp. 134–143).

Finally, the analysis of B cells and NK in our study did not reveal a significant reduction in their percentages compared to the controls. NK cells may be sensitive to events occurring in between the pre- and post-dialysis. However, previous studies showing the characteristics of NK cells in HD patients have led to conflicting results because of limited study sizes [24] (pp. 359–393). The reduced number of NK cells and their impaired function might enhance the susceptibility to viral infections in patients with ESKD, resulting from decreased killing of infected cells and transformed cells [5] (pp. 82–94). It is known that ESKD patients show an increase in monocytes and macrophages expressing Toll-like receptors molecules 2 and 4 (TLR-2, TLR4), suggesting an activation of these cells in response to HD [25] (pp. 247–254). A different study demonstrated a decrease and dysfunction in dendritic cells in HD patients compared to healthy controls [26] (pp. 737–746). Additionally, several alterations related to complement activation have been described during HD [27] (pp. 1143–1157). Regarding the innate immune response, compared to healthy subjects, the expression of surface antigens, including CD28, CD69 and CD25, that play a pivotal role in immune cell activation, is reduced in HD patients, leading to an impairment of CD4^+^ stimulation and proliferation [28] (pp. 189–200). Moreover, CD4^+^ cells derived by HD subjects had an increased stimulation of the Th1 subset, resulting in an increased secretion of interferon gamma (IFN-ꙋ), confirming the pro-inflammatory activity of HD treatment [29].

## 5. Conclusions

The present study provides an overview of the impact of a single hemodialysis treatment on immune system subpopulations in a homogeneous group of patients. The results obtained highlighted the alterations caused by the exposition to dialysis filters on the major immune system subsets and could help in understanding the impact of dialysis during chronic treatment.

## Figures and Tables

**Table 1:** Baseline characteristics and inflammatory parameters of the patients. Values are expressed as medians (q25–q75). One-way ANOVA was used to calculate the *p* value.

Characteristics	Filtryzer BGU1.6 (N = 12)	Theranova (N = 12)	HFR17 (N = 11)	*p* Value
Age (years)	74 (54–88)	74 (56–86)	76.5 (55.2–87.3)	0.989
Women, n (%)	6 (50)	7 (58.3)	6 (54.5)	0.925
BMI (Kg/m^2^)	22.22 (19.87–28.11)	27.9 (22.15–28)	24.39 (23.18–32.5)	0.74
Neutrophils (10^9^/L)	4.3 (3.5–5.5)	4.4 (3.6–7.3)	4.3 (3.4–5.3)	0.939
Lymphocytes (10^9^/L)	1.2 (0.9–1.6)	1.2 (0.9–1.5)	1.18 (0.9–1.4)	0.945
Platelets (10^9^/L)	170 (105–227)	168 (99.7–224.5)	170 (101.5–229)	0.983
Ferritin (ng/mL)	150 (60.7–272.5)	149.5 (66–319)	149.5 (63.2–248)	0.952
Transferrin saturation (mg/mL)	25.7 (12.8–37.2)	25.5 (13–37.7)	25.5 (13.4–38)	0.976
Serum iron (µg/dL)	41 (27.7–78)	51 (30–78)	47 (28.5–76.5)	0.921
Albumin (g/dL)	3.6 (3.3–3.8)	3.5 (3.4–4.2)	3.5 (3.4–3.9)	0.986
NLI	3.8 (2.8–5)	3.6 (2.9–5)	3.7 (2.9–4.87)	0.983
PLI	134 (100–236.5)	120.7 (89–233)	134.3 (97.2–230.7)	0.970
CRP (mg/dL)	0.35 (0.07–6.1)	0.6 (0.21–0.8)	0.15 (0.08–0.2)	0.942
Iron therapy (%)	33.33	58.33	36.36	/
EPO therapy (%)	100	100	100	/

BMI: body mass index; NLI: neutrophil-lymphocyte ratio; PLI: platelet-lymphocyte ratio. CRP: C Reactive Protein; EPO: erythropoietin.

**Table 2:** Dialysis adequacy. Values are expressed as median (q25–q75). *p* values were evaluated using one-way ANOVA.

Characteristics	Filtryzer BGU1.6 (N = 12)	Theranova (N = 12)	HFR17 (N = 11)	*p* Value
Time spent on dialysis (months)	47 (19.5–60)	44 (17.5–60)	44.5 (15.5–60)	0.903
Type of dialysis (stHD/HDF)	9/3	12/0	0/11	/
Vascular access (AVF/CVC)	6/6	5/7	8/3	/
Blood flow (mL/min)	300 (270–300)	300 (275–300)	300 (280–300)	0.967
Dialysis duration (min)	240 (240–240)	240 (240–240)	240 (240–240)	0.809
Session per week	3	3	3	/
V men (L)	17.8 (17–21.3)	17.03 (15.1–18.9)	18.6 (17.3–20.1)	0.922
V Women (L)	13.3 (12.3–14.3)	14.34 (13.3–16.1)	17.8 (15.3–23.9)	0.934
Phosphorous (mg/dL)	4.65 (3.85–6.38)	5.1 (3.9–6.2)	5.5 (4.35–6.08)	0.810
PTH	305 (145.8–546.8)	300 (116–473)	314 (190–340)	0.72
Calcium (mg/dL)	8.9 (8.08–9.5)	8.3 (7.9–8.8)	8.8 (8.73–9.22)	0.881
Delta on dry weight	3.1 (2.07–4.05)	2.2 (2–2.7)	3.1 (2–3.5)	0.69

stHD: standard hemodialysis; HDF: online hemodiafiltration; AVF: arteriovenous fistula; CVC central venous catheter; V: urea distribution; PTH: parathyroid hormone.

**Table 3:** Dialysis efficiency parameters. Values are expressed as median (q25–q75). *p* values were evaluated using one-way ANOVA. B2M: beta 2 microglobulin; k: dialyzer clearance of urea; t: dialysis time; V: urea volume.

Characteristics	Filtryzer BGU1.6 (N = 12)	Theranova (N = 12)	HFR17 (N = 11)	*p* Value
Urea (mg/dL)	141 (83–152.5)	122 (83–165)	112.5 (98.5–137.8)	0.673
B2M	34.5 (27.8–33.4)	29.9 (27.85–33.4)	31.2 (25.3–42.6)	0.864
Potassium (mEq/L)	4.9 (4.35–5.15)	4.5 (4.3–5.7)	5.95 (5.28–6.08)	0.791
Kappa chains (mg/L)	144.6 (118.2–224.8)	165.3 (110.1–211.6)	215 (123–301.4)	0.513
Lamda chains (mg/L)	127.2 (62.75–164.5)	133.6 (94.8–138.3)	121.3 (105.7–142)	0.786
Global kt/V	1.58 (14.83–1.68)	1.64 (1.52–1.67)	1.66 (1.51–1.74)	0.834

## Data Availability

Not applicable.
